# Would the width of a metal rib spreader affect postoperative pain in patients who undergo video-assisted mini-thoracotomy (VAMT)?

**DOI:** 10.3389/fonc.2022.1039737

**Published:** 2022-10-26

**Authors:** Linlin Wang, Lihui Ge, Ninghua Fu, Yi Ren

**Affiliations:** ^1^ Department of Thoracic Surgery, Shenyang Chest Hospital & Tenth People’s Hospital, Shenyang, Liaoning, China; ^2^ Department of Health Management, Shengjing Hospital of China Medical University, Shenyang, Liaoning, China; ^3^ Department of Thoracic Surgery, The Affiliated Hospital of Guizhou Medical University, Guiyang, Guizhou, China

**Keywords:** lung cancer, lobectomy, video-assisted mini-thoracotomy (VAMT), metal rib spreader, visual analogue scale pain scores

## Abstract

**Background:**

Hitherto, no study has evaluated postoperative pain in patients with non-small cell lung cancer (NSCLC) treated with video-assisted mini-thoracotomy (VAMT). In this study, we aimed to assess postoperative pain related to the width of the metal rib spreader in patients who underwent lobectomy using VAMT.

**Methods:**

We retrospectively analysed the data of 94 consecutive patients with NSCLC who underwent lobectomy using VAMT at our institution between March 2019 and May 2022. We divided the patients into groups according to the width ratio of the rib spreader to that of a single intercostal space. Patients with width ratios ≤ 2.5 times were assigned to group A, and those with width ratios > 2.5 times were assigned to group B. Pre-, intra-, and postoperative data were collected and reviewed.

**Results:**

We successfully performed VAMT in 94 patients with NSCLC. Forty-five patients were in group A, and 49 were in group B. There were no intraoperative mortalities, although one patient, due to respiratory failure, experienced 30-day mortality. There were no significant differences between the two groups in terms of the blood loss volume, operative time, drainage time, postoperative complications, length of hospital stay, or number of lymph node stations explored and retrieved. The drainage volumes (Day 1–Day 3) were higher in group B than in group A (*P* < 0.05). The postoperative visual analogue scale (VAS) pain scores were significantly lower in Group A than in Group B at 12, 24, and 48 h (*P* < 0.05), although there was no significant difference in the VAS scores between the two groups at 72 h and 1 week postoperatively (*P* > 0.05).

**Conclusion:**

The smaller the width of the metal rib spreader used in surgery, the less pain experienced by the patient and the faster the recovery. Multicentre, randomised, controlled trials should be conducted in the future.

## Introduction

Anatomic resection is the current treatment of choice for non-small-cell lung cancer (NSCLC). With the advancement of minimally invasive techniques, thoracoscopic surgery has become the mainstream treatment for early-stage lung cancer (1). Thoracoscopic surgery has the advantages of inducing less trauma, leading to less postoperative pain, and resulting in a faster recovery time than traditional thoracotomy (2, 3). However, thoracoscopic surgery is difficult and risky in cases involving total pleural adhesion, unclear separation of hilar organs, enlarged hilar lymph nodes, tumour invasion of important vessels, or intraoperative bleeding and if conversion to thoracotomy is required intraoperatively (4–6). Interestingly, if the small incision is assisted by thoracoscopy, the potential or existing difficulties of the surgery may be minimised. Rib dissection is not required in such cases; however, the postoperative pain associated with the use of a rib spreader is unknown. This study aimed to evaluate the effect of the width of the metal rib spreader used in surgery on postoperative pain and perioperative efficacy of video-assisted mini-thoracotomy (VAMT) lobectomy.

## Materials and methods

### Patients

We retrospectively analysed the data of 94 patients with lung cancer who underwent lobectomy *via* VAMT between March 2019 and May 2022 at our hospital. Seven patients received neoadjuvant therapy before the surgery. The inclusion criteria were the following: 1) pathological diagnosis of NSCLC; 2) stage I–IIIA (Tumour [T], Node [N], Metastasis [M] stages: T1–4N0–2M0); 3) American Society of Anesthesiologists classification: I–II; 4) complete visual analogue scale (VAS) scoring data; and 5) no surgical contraindications. The exclusion criteria were as follows: 1) incomplete survival or clinical data; 2) diagnosis of small cell lung cancer; 3) a history of thoracotomy; 4) long-term use of pain medication; 5) ropivacaine allergy; 6) abnormal coagulation function; and 7) severe cardiopulmonary dysfunction. In the event of accidental intraoperative haemorrhage due to large blood vessel injury, the presence of extensive thoracic adhesions, or dense adhesion of hilar lymph nodes, video-assisted thoracoscopic surgery (VATS) was converted to VAMT, regardless of the size of the nodule. We divided the patients into groups according to the width ratio of the metal rib spreader to that of a single intercostal space. Patients with width ratios ≤ 2.5 times were assigned to group A, and those with width ratios > 2.5 times were assigned to group B. Pre-, peri-, and postoperative patient details and outcome variables were collected by means of patient enquiry and clinical assessment. Postoperative chest radiographs were reviewed to determine whether there was any abnormality in the thoracic cavity.

### Pain management

Pain management was standardised for all patients in both groups. Postoperative analgesics were administered by a nurse specialising in pain management.

Before closing the chest cavity in each patient, an intercostal nerve block technique was used. More specifically, the puncture point of the nerve block was near the intercostal vessels and 2 cm lateral to the costal joint. A puncture was made from the parietal pleura to the outside. Subsequently, a fine needle was used for vertical insertion into the upper edge of the rib. If no blood was withdrawn, 3 mL of 0.375% ropivacaine (Naropin, AstraZeneca AB, Sweden) and 0.001% adrenaline was injected into a single intercostal space, filling the pleura around the intercostal nerves. At the end of the surgery, 3 mL of 0.375% ropivacaine was administered to infiltrate the wound.

Postoperative use of opioid analgesics was documented in the nursing records. Opioids (5 mg dezocine intramuscular injection every 3–6 h, if necessary) were administered upon request if the patient was in severe pain. Patients with VAS pain scores ≥ 4 (Day 1–Day 3) were treated twice daily with 30 mg of intravenous non-steroidal anti-inflammatory drugs (ketorolac tromethamine, Shandong New Times Pharmaceutical Co., China).

### Surgical technique

**Figure 1:**
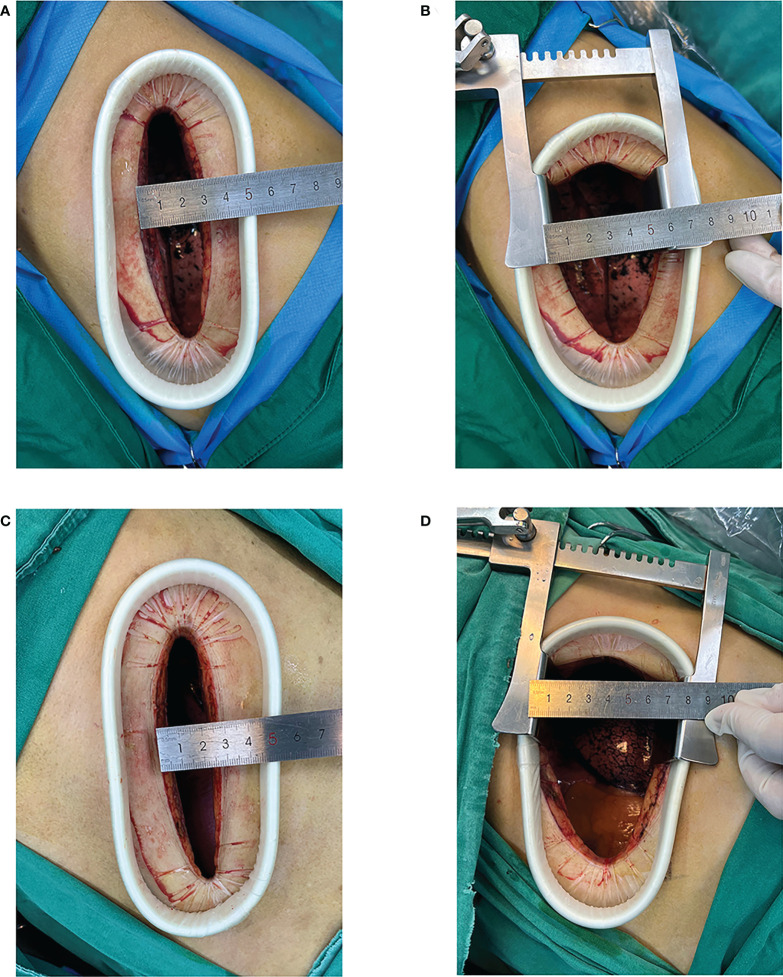
Video-assisted mini-thoracotomy (VAMT) surgical incision. (Top row) Group A: original intercostal incision **(A)**; intercostal distance measured after using the metal rib spreader **(B)** (Bottom row) Group B: original intercostal incision **(C)**; intercostal distance measured after using the metal rib spreader **(D)**.

We used general anaesthesia with single-lung ventilation for the VAMT lobectomy, which was accomplished using a double-lumen endotracheal tube. The patient was placed in a full lateral decubitus position, and the operation was performed as follows: an incision of approximately 8–10 cm was made in the fourth or fifth intercostal space at the anterior edge of the latissimus dorsi without cutting the ribs. A plastic wound protector was placed in the incision, and a metal rib spreader was used for assistance (**Figure 1**). We used multiple surgical instruments during the procedure in the same incision, such as a 30° thoracoscope, an Echelon Flex 45 (Ethicon Endo-Surgery, LLC, USA), and a long, curved endoscopic surgical instrument with double articulation and curved suction. The perihilar arteries, veins, and bronchi were dissected using thoracoscopy. Freed lung fissures and pleural adhesions, and mediastinal lymphadenectomy was performed. We used the Echelon Flex 45 endostapler to manage the bronchus, incomplete fissures, and the main blood vessels. An intercostal nerve block was performed under thoracoscopic assistance using specialised long-needled instruments.

### VAS pain scores

The intensity of postoperative pain was determined using VAS scoring (7). The VAS was based on a 10-point scale ranging from ‘no pain’ (VAS = 0) to ‘extremely painful’ (VAS = 10). Patients were asked to mark a position on the scale that corresponded to the pain they were experiencing and to record the score accordingly. Nursing staff recorded the postoperative pain scores of patients at 12, 24, 48, and 72 h and 1 week.

### Statistical analyses

Continuous variables are expressed as the mean ± standard deviation, whereas categorical variables are presented as percentages. Continuous variables were compared between groups *via* Student’s *t*-test, whereas Pearson’s chi-square test or Fisher’s exact test was used to compare categorical variables between groups. IBM Statistical Package for the Social Sciences for Windows software, version 26 (IBM Corp., Armonk, N.Y., USA) was used to analyze the data. Statistical significance was set at *P* < 0.05 (two-sided). Figures were generated using GraphPad Prism software (version 8.3.1, GraphPad Software Inc, California, USA).

## Results

### Patient characteristics

Ninety-four patients underwent lobectomy *via* VAMT between March 2019 and May 2022. The study included 45 patients in group A and 49 in group B. There were no intraoperative mortalities; however, one patient died within the first 30 days due to respiratory failure. Preoperative intervention with neoadjuvant therapy was performed in seven patients. Age, sex, pulmonary function, arterial blood gas analysis, laterality, incision location, and lobectomy characteristics were not significantly different between the two groups (*P* > 0.05). Patient characteristics are presented in **Table 1**. Chest radiography revealed no obvious thoracic deformity in any case.

**Table 1:** Baseline patient characteristics.

Characteristic	Group A (n = 45)	Group B (n = 49)	Total (n =94)	*P* value
Age (years)				0.109^**^
≤ 60 (%)	22 (48.89)	16 (32.65)	38 (40.43)	
> 60 (%)	23 (51.11)	33 (67.35)	56 (59.57)	
Mean ± SD	60.31 ± 7.42	61.27 ± 8.70	60.81 ± 8.08	0.570^*^
Sex (%)				0.473^**^
Female	14 (31.11)	12 (24.49)	26 (27.66)	
Male	31 (68.89)	37 (75.51)	68 (72.34)	
Pulmonary function (Mean ± SD)				
FEV_1_	86.14 ± 15.60	84.74 ± 19.13	85.41 ± 17.45	0.700^*^
FVC	87.04 ± 17.25	87.41 ± 16.96	87.23 ± 17.01	0.915^*^
MVV	80.24 ± 14.01	82.08 ± 19.72	81.20 ± 17.16	0.606^*^
Arterial blood gas analysis (Mean ± SD)				
PAO_2_ (mmHg)	79.56 ± 7.22	81.43 ± 10.76	80.53 ± 9.24	0.329^*^
PACO_2_ (mmHg)	41.71 ± 3.69	41.51 ± 3.44	41.61 ± 3.55	0.785^*^
SAO_2_ (%)	96.00 ± 1.00	95.76 ± 1.55	95.87 ± 1.31	0.369^*^
Laterality (%)				0.808^**^
Left	25 (55.56)	26 (53.06)	51 (54.26)	
Right	20 (44.44)	23 (46.94)	43 (45.74)	
Incision location (%)				0.096^**^
Fourth intercostal space	15 (33.33)	9 (18.37)	24 (25.53)	
Fifth intercostal space	30 (66.67)	40 (81.63)	70 (74.47)	
Nodule location (%)				
Left upper lobe	16 (35.56)	17 (34.69)	33 (35.11)	0.930^**^
Left lower lobe	9 (20.00)	9 (18.37)	18 (19.15)	0.841^**^
Right upper lobe	8 (17.78)	9 (18.37)	17 (18.09)	0.941^**^
Right middle lobe	4 (8.89)	4 (8.16)	8 (8.51)	1.000^***^
Right lower lobe	8 (17.78)	10 (20.41)	18 (19.15)	0.746^**^
Lobectomy (%)				
Left upper	16 (35.56)	17 (34.69)	33 (35.11)	0.930^**^
Left lower	9 (20.00)	9 (18.37)	18 (19.15)	0.841^**^
Right upper	8 (17.78)	9 (18.37)	17 (18.09)	0.941^**^
Right middle	3 (6.67)	1 (2.04)	4 (4.26)	0.346***
Right middle and lower	4 (8.89)	7 (14.29)	11 (11.70)	0.416^**^
Right lower	5 (11.11)	6 (12.24)	11 (11.70)	0.864^**^

SD, standard deviation; FEV_1_, forced expiratory volume in 1 s; FVC, forced vital capacity; MVV, maximal ventilatory volume; PAO_2_ partial pressure of oxygen in the artery; PACO_2_ partial pressure of carbon dioxide in the artery; SAO_2_ arterial oxygen saturation.

P value: * Student’s t-test; ** Pearson’s chi-square test; *** Fisher’s exact.

### Surgical outcomes

No significant differences were found in terms of the volume of blood loss, operative times, drainage times, incidence of postoperative complications, length of hospital stay, or number of lymph node stations explored and retrieved (*P* > 0.05). The drainage volume was higher in group B than in group A for all three days (Day 1: 336.22 ± 114.02 vs 458.78 ± 209.91; Day 2: 322.78 ± 116.05 vs 403.37 ± 216.38; Day 3: 239.78 ± 112.76 vs 319.69 ± 233.48; *P* < 0.05). The postoperative pathologic diagnoses and pathologic stages are shown in **Table 2**. Postoperative complications occurred in 7 (15.56%) and 11 (22.45%) patients in groups A and B, respectively (**Table 2**).

**Table 2:** Perioperative results.

Characteristic	Group A (n = 45)	Group B (n = 49)	Total (n = 94)	*P* value
Surgical results (Mean ± SD)				
Operative time (min)	204.89 ± 66.71	222.51 ± 75.52	214.07 ± 71.61	0.235^*^
Blood loss volume (mL)	211.33 ± 180.66	241.22 ± 216.47	226.91 ± 199.63	0.471^*^
Drainage time (days)	8.36 ± 4.80	9.18 ± 5.61	8.79 ± 5.23	0.446^*^
Drainage volume (mL)				
Day 1	336.22 ± 114.02	458.78 ± 209.91	400.11 ± 180.78	0.001^*^
Day 2	322.78 ± 116.05	403.37 ± 216.38	364.79 ± 179.38	0.029^*^
Day 3	239.78 ± 112.76	319.69 ± 233.48	281.44 ± 189.11	0.040^*^
Day 4	219.11 ± 112.73	258.98 ± 134.62	239.89 ± 125.56	0.125^*^
Length of hospital stay (days)	11.56 ± 5.68	12.53 ± 6.40	12.06 ± 6.05	0.438^*^
Number of nodal stations explored	9.22 ± 1.92	8.86 ± 1.43	9.03 ± 1.68	0.295^*^
Number of lymph nodes retrieved	17.38 ± 6.38	17.63 ± 6.90	17.51 ± 6.62	0.853^*^
Tumour (T)-stage (%)				
T1	8 (17.78)	14 (28.57)	22 (23.40)	0.217^**^
T2	17 (37.78)	17 (34.69)	34 (36.17)	0.756^**^
T3	9 (20.00)	10 (20.41)	19 (20.21)	0.961^**^
T4	11 (24.44)	8 (16.33)	19 (20.21)	0.328^**^
Node (N)-stage (%)				
N0	26 (57.78)	32 (65.31)	58 (61.70)	0.453^**^
N1	10 (22.22)	7 (14.29)	17 (18.09)	0.318^**^
N2	9 (20.00)	10 (20.41)	19 (20.21)	0.961^**^
Tumour stage (AJCC^a^ eighth ed.) (%)				
I	6 (13.33)	13 (26.53)	19 (20.21)	0.111^**^
II	21 (46.67)	18 (36.73)	39 (41.49)	0.329^**^
III	17 (37.78)	18 (36.73)	35 (37.23)	0.917^**^
IV^b^	1 (2.22)	0 (0.00)	1 (1.06)	0.479^***^
Pathological types (%)				
Adenocarcinoma	18 (40.00)	20 (40.82)	38 (40.43)	0.936^**^
Squamous cell carcinoma	23 (51.11)	24 (48.98)	47 (50.00)	0.836^**^
Others	4 (8.89)	5 (10.20)	9 (9.57)	1.000^***^
Postoperative complications (%)				
Prolonged air leak	1 (2.22)	0 (0.00)	1 (1.06)	0.479^***^
Subcutaneous emphysema	4 (8.89)	7 (14.29)	11 (11.70)	0.416^**^
Haemothorax	1 (2.22)	2 (4.08)	3 (3.19)	1.000^***^
Atelectasis	0 (0.00)	1 (2.04)	1 (1.06)	1.000^***^
Bronchopleural fistula	0 (0.00)	1 (2.04)	1 (1.06)	1.000^***^
Cardiac arrhythmias	1 (2.22)	0 (0.00)	1 (1.06)	0.479^***^

a AJCC, American Joint Committee on Cancer.

b Postoperative pathological diagnosis of lung cancer with pleural metastasis.

P value: ^*^ Student’s t-test; ^**^ Pearson’s chi-square test; ^***^ Fisher’s exact test.

### VAS pain scores

**Figure 2:**
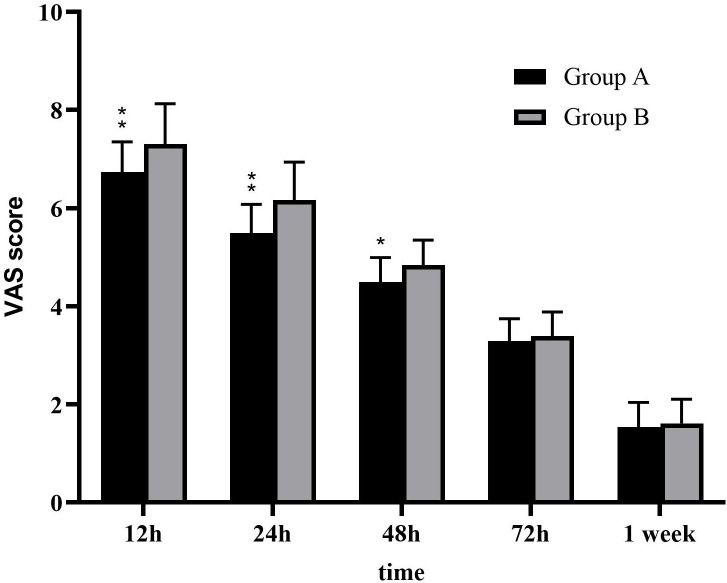
Postoperative visual analogue scale (VAS) pain scores. The postoperative pain scores in group B after surgery were significantly higher compared with that in group A (*P* < 0.05).**:*P* < 0.001 *:*P* < 0.05.

Postoperative pain was measured using VAS scores. The mean postoperative VAS scores of group A at 12, 24, 48, and 72 h and 1 week were 6.73 ± 0.62, 5.49 ± 0.59, 4.49 ± 0.51, 3.29 ± 0.46, and 1.53 ± 0.51, respectively. Meanwhile, the mean postoperative VAS scores of group B at 12, 24, 48, and 72 h and 1 week were 7.31 ± 0.82, 6.16 ± 0.77, 4.84 ± 0.51, 3.39 ± 0.49, and 1.61 ± 0.49, respectively. In contrast to group B, group A achieved significantly lower postoperative VAS scores at 12, 24, and 48 h (*P* < 0.05) (**Figure 2**). However, there were no significant differences in incision location and laterality with respect to the postoperative VAS scores at 12, 24, 48, and 72 h and 1 week between the two groups (*P* > 0.05).

## Discussion

Surgical lobectomy with mediastinal lymph node dissection is the standard of care for resectable NSCLC (8, 9). Most traditional surgical methods involve anterolateral incisions, which are long incisions that lead to a higher blood loss volume, take a longer time to open and to close the chest, and occasionally require the surgeon to cut through the ribs (10). Therefore, the application of thoracoscopic surgery has greatly reduced postoperative complications and accelerated recovery for patients who experience obvious postoperative pain, have large incision scars, exhibit slow postoperative healing, and have a high incidence of complications (11). In recent years, with the advancement of endoscopic technology and instruments, minimally invasive surgery has evolved from traditional three-port and two-port to uniportal VATS (12–14). Non-intubated and subxiphoid uniportal VATS and robot-assisted thoracic surgery are emerging in real-world clinical scenarios (15–17).

However, regardless of whether a nodule is < 1 cm, VAMT may be required in cases in which there is preoperative suspicion of hilar lymph node metastasis or in patients with complete pleural adhesion, unclear hilar organ separation, tumour invasion of important vessels, and intraoperative bleeding, among other factors. This approach represents a compromise between traditional thoracotomy and VATS. VAMT has certain advantages, including the following: 1) as VAMT is a direct vision procedure, thoracoscopy can be used to explore hidden locations that cannot be directly observed otherwise; 2) the ability to insert multiple instruments in the same incision is convenient for surgery and shortens the operation time while reducing the risk and simultaneously helping to achieve oncological effects; and 3) the surgical incision in VAMT is smaller than that for conventional thoracotomy, thereby reducing the damage to the pectoralis major, latissimus dorsi, and other muscle groups. The incision protector wraps around the intercostal muscles to reduce bleeding and potential damage to the muscles. Tsunezuka et al. reported that among the 34 patients who underwent surgery using a wound retraction system for lung resection by VATM, none experienced severe postoperative chest pain, wound infection, or contamination (18). Therefore, we recommend the routine use of incision protectors for VAMT.

Traditional open surgery requires rib spreading, whereas VAMT does not. As a result, patients experience less postoperative pain and fewer chest wall deformities. Furthermore, the range of the metal rib spreader’s width is limited compared with that of traditional thoracotomy; therefore, postoperative recovery is faster and patient satisfaction tends to be higher. However, Ichimura et al. believed that there was a negligible impact on postoperative quality of life and pain in patients undergoing lobectomy through VAMT, with or without the use of a metal spreader (19). With the increasing maturity of minimally invasive technologies and progressive improvements in operating instruments, we suggest that experienced surgeons should consider not using a metal rib spreader in VAMT; however, when a metal rib spreader is used, it should have a width ratio not exceeding 2.5 times the width of a single intercostal space.

In recent years, an increasing number of studies have investigated the postoperative quality of life and postoperative pain in patients with lung cancer (20, 21). Postoperative pain not only brings great discomfort to patients, but it also leads to changes in respiratory and circulatory function, immune function, and other complications such as pneumonia, atelectasis, and even chronic pain; these changes can seriously affect the patient’s functional recovery, treatment effects, and quality of life (22, 23). The effect that the width of expansion of a metal rib spreader can have on postoperative pain was previously unclear; therefore, we performed a retrospective analysis. The width of the metal rib spreader was < 2.5 times the width of the intercostal distance, and the pain score in group A was lower than that in group B within the first 48 h after surgery (*P* < 0.05), as was the daily drainage volume (*P* < 0.05). However, there were no significant differences in the postoperative drainage time or duration of hospitalisation between the two groups. We also compared the difference in postoperative pain between patients with different intercostal incision sites (fourth or fifth intercostal space), although there was no significance between the two groups (*P* > 0.05).

The common causes of perioperative pain in patients undergoing thoracic surgery include surgical trauma, placement of the indwelling drainage tube, pleural injury, intercostal nerve injury or compression, postural discomfort, and anxiety or tension, among other factors (24). Nociceptive stimulation caused by thoracic surgery, including that caused directly by the incision or subsequent release of inflammatory factors (25), can activate peripheral and central nervous system neurons leading to nociceptive sensitisation, which can then cause acute postoperative pain in patients. If acute pain is not well-controlled, it can cause a decline in neuroregulatory function and further peripheral nerve terminal sensitisation and central nervous system sensitisation, leading to the development of chronic pain in some patients (26). Therefore, in real-world clinical scenarios, some patients who have received VATS complained of postoperative pain lasting for a long period of time before any improvement, sometimes for more than half a year. The factors contributing to the diversity of pain outcomes in such patients remain to be further investigated. Although postoperative analgesia is very important in promoting early recovery and reducing the incidence of postoperative complications, this study only compared perioperative pain without long-term follow-up. In the future, multicentre, randomised, controlled trials should be conducted to further investigate the contributing factors responsible for the diversity of pain-related outcomes in patients. The treatment mode involving preoperative neoadjuvant therapy combined with surgery for patients with locally advanced NSCLC is becoming increasingly popular in clinical practice (27). Notably, the safety and feasibility of thoracoscopic surgery after preoperative neoadjuvant therapy are controversial because of the appearance of diffuse fibrosis in lung tissue after neoadjuvant therapy, intrathoracic tissue adhesion, increased vascular fragility, severe thoracic fibrosis and tissue oedema, insufficient structural space, and fusion of hilar lymph nodes, among other complications, resulting in certain surgical difficulties (28, 29). Dell’Amore et al. (30) analysed 155 patients with stage IIA–IIIB NSCLC who underwent VATS or thoracotomy after neoadjuvant chemotherapy. The operation time, number of drainage days, pain score at discharge, and duration of the hospital stay were significantly better in the thoracotomy group than in the VATS group (*P* < 0.01). Furthermore, Zhao et al. (31) studied the safety and feasibility of epidermal growth factor receptor-tyrosine kinase inhibitor induction therapy versus chemotherapy followed by lung resection in patients with stage IIIA–N2 lung adenocarcinoma; they found that there were no significant differences in surgical indicators between the two groups. Additionally, Bott et al. (32) reported the preliminary surgical results of the Check-Mate-159 trial, in which 20 patients with stage I–IIIA NSCLC underwent surgery and 14 underwent thoracotomy. Among them, seven were transferred to receive thoracotomy because of serious adhesions between important blood vessels, lung tissues, lymph nodes, and surrounding tissues. In our study, seven patients who underwent a thoracoscopic-assisted small incision procedure also underwent preoperative neoadjuvant therapy; in those cases, intraoperative tissue vascular adhesions were found to significantly increase the complexity of the operation. However, thoracoscopy-assisted small incision not only reduces the risk of surgery but also achieves oncological effects. Therefore, we recommend thoracoscopic small incision surgery in patients undergoing preoperative neoadjuvant therapy. A multicentre, randomised, controlled trial with a large sample size is needed to explore the surgical modalities of patients receiving preoperative neoadjuvant therapy for lung cancer.

This study had some limitations. First, this was a single-centre, retrospective study with selection and information bias. Second, there was a lack of information pertaining to the effects of VAMT on immune function, inflammatory stress responses, and hemodynamic stability of the body during and after surgery. Third, there are differences in the analgesic effects of different local anaesthetics, which may have biased the postoperative pain scores. Fourth, this study only compared acute perioperative pain without a long-term follow-up period. Additionally, the VAS pain scores used for pain assessment are subjective. Therefore, prospective studies with additional evidence need to be conducted in future.

## Conclusion

The smaller the width of the metal rib spreader used in surgery, the less pain the patient will experience and the faster the recovery will be. Furthermore, we found that patients who underwent preoperative neoadjuvant therapy were more suitable for VAMT. However, multicentre, randomised, controlled trials should be conducted in the future.

## Data availability statement

The original contributions presented in the study are included in the article/Supplementary Material. Further inquiries can be directed to the corresponding author.

## Author contributions

LW drafted the manuscript. Data acquisition was performed by LW and LG. LW and NF designed the analysis. YR and LW conceived and designed the study. All authors have read and approved the final manuscript. All authors contributed to the article and approved the submitted version.

## Funding

This study was supported by Liaoning Province Science and Technology Program Project (2019JH8/10300089). The funding agency did not participate in the study design, data collection, analysis, or interpretation and was not involved in writing the manuscript.

## Conflict of interest

The authors declare that the research was conducted in the absence of any commercial or financial relationships that could be construed as a potential conflict of interest.

## Publisher’s note

All claims expressed in this article are solely those of the authors and do not necessarily represent those of their affiliated organizations, or those of the publisher, the editors and the reviewers. Any product that may be evaluated in this article, or claim that may be made by its manufacturer, is not guaranteed or endorsed by the publisher.
